# Comparison of the Genetic Diversity of the Captive and Wild Populations of the Tsushima Leopard Cat Using a GRAS-Di Analysis

**DOI:** 10.3390/ani12111464

**Published:** 2022-06-05

**Authors:** Hideyuki Ito, Nobuyoshi Nakajima, Manabu Onuma, Miho Inoue-Murayama

**Affiliations:** 1 Kyoto City Zoo, Kyoto 606-8333, Japan; 2 Wildlife Research Center, Kyoto University, Kyoto 606-8203, Japan; mmurayama@wrc.kyoto-u.ac.jp; 3 Biodiversity Division, National Institute for Environmental Studies, Tsukuba 305-8506, Japan; naka-320@nies.go.jp (N.N.); monuma@nies.go.jp (M.O.)

**Keywords:** Tsushima leopard cat, GRAS-Di, genome-wide analysis, conservation program

## Abstract

**Simple Summary:**

The Tsushima leopard cat (*Prionailurus bengalensis euptilurus*) is exclusively found on the Tsushima Island in Japan; however, its population has been declining in recent years and is in danger of extinction. Hence, effort is underway for both in situ and ex situ conservation. Genetic management is also important in the management of captive populations, and various studies are being conducted regarding the same. In our previous study, we developed a reference genome of the Tsushima leopard cat and used GRAS-Di analysis, a genome-wide analysis, to genetically evaluate the wild populations. In this study, we attempted to improve the Tsushima leopard cat reference genome and compared the captive and wild populations by GRAS-di analysis. The results showed that the captive population had almost the same genetic diversity as the wild population and managed to remain in good condition.

**Abstract:**

The Tsushima leopard cat (*Prionailurus bengalensis euptilurus*) (TLC) is a regional population of the Amur leopard cat (*P. bengalensis euptilurus*) that lives only on the Tsushima Island in Japan and is threatened with extinction. Because the TLC population is small, genetic management is important. In this study, we obtained the draft genome of the TLC and identified single-nucleotide polymorphism (SNP) markers using a genotyping by random amplicon sequencing-direct (GRAS-Di) analysis. We genotyped 31 captive individuals and 50 wild individuals, of which 48 were from a previous study. The identified SNPs were used to clarify the genetic diversity and genetic structure of the wild and captive populations of the TLC. The size of the genome was estimated to be about 2.42 Gb. The number of SNP markers developed was 139, and although *PID* and probability of exclusion obtained using these SNP markers were not as high as those reported in the studies of other wild species, these SNP markers could be used to identify individuals and parentage. Moreover, the genetic diversity indices of the captive population were similar to those of the wild population. These SNP markers will be useful for understanding the ecology of the TLC and planning conservation strategies.

## 1. Introduction

**Figure 1:**
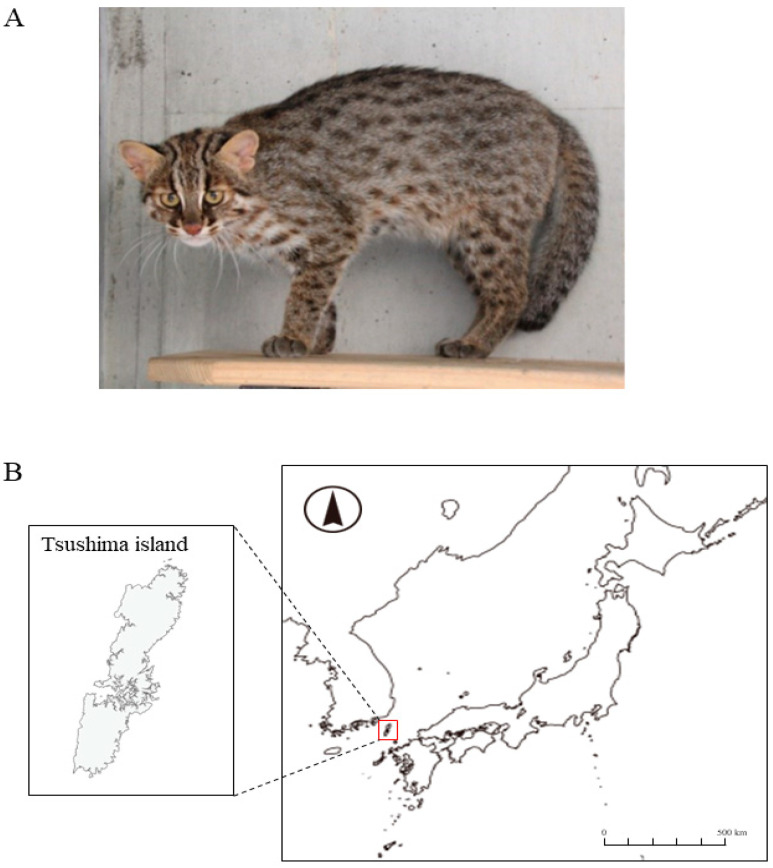
(**A**) Photograph of Tsushima leopard cat, (**B**) Location of Tsushima Island.

The Tsushima leopard cat (TLC) (*Prionailurus bengalensis euptilurus*) is a regional population of the Amur leopard cat (*P. bengalensis euptilurus*) that is found only in Tsushima, Japan (Figure 1). This species is declining because of habitat fragmentation, depletion of feeding grounds, and road killing. The population in the wild is estimated to comprise 90–100 animals; moreover, the TLC has been classified as a critically endangered species in the Japanese Red List. In 1995, the Japanese Ministry of the Environment established a program promoting “the rehabilitation of natural habitats and maintenance of viable populations” for the TLC. As a part of this program, the Japanese Ministry of the Environment, in cooperation with the Japanese Association of Zoos and Aquariums and other organizations, conducted an ex situ breeding program.

For several endangered species, captive breeding programs have been established with the primary goal of maintaining the genetic diversity of target populations, avoiding the negative effects of inbreeding, and maintaining their adaptive potential [1,2]. The most common target of genetic management is to retain 90% of the genetic diversity for 100 years [1,3]. Traditionally, genetic management is based on pedigree information; however, there can be shortcomings in this method, such as unknown kinship between founders, missing data, etc. These disadvantages lead to the possibility of an overestimation of genetic diversity [4,5,6]. Recently, to resolve these issues, molecular genetic analyses were integrated with traditional pedigree analyses in some captive breeding programs [7,8,9,10,11,12]. The integrated method can augment the genetic information solely based on pedigree data and result in better genetic management plans.

Recent advances in molecular genetic techniques have enabled rapid and large-scale development of DNA markers [2]. Additionally, more economical and efficient approaches have been developed, such as restriction site-associated DNA sequencing (RADSeq) [13], double-digested RADSeq (ddRADSeq) [14], multiplexed ISSR genotyping by sequencing (MIG-Seq) [15], and genotyping by random amplicon sequencing-direct (GRAS-Di) [16]. In our previous study [2], we developed the draft genome of the TLC using only short-read sequencing data and evaluated the genetic diversity of the wild populations of TLC by GRAS-Di analysis. In the process of developing SNP markers, the most efficient method was to use the TLC genome as a reference genome [2]. It is expected that the improved accuracy of the reference genome will increase the number of mapped reads, leading to the development of more SNP markers.

In this study, to assess the genetic diversity of the captive population of TLC, we modified the draft genome using short-read and long-read sequencing data and compared the genetic diversity of captive and wild populations of TLC by GRAS-Di analysis.

## 2. Materials and Methods

### 2.1. Sample Collection

This study was conducted in strict accordance with the guidelines for ethics in animal research established by the Wildlife Research Center of Kyoto University. The Kyoto City Zoo approved the research (#20170426). We obtained blood samples from 31 TLCs in captivity and muscle samples from 2 TLCs that died in the wild (i.e., founders). The samples of the captive population included five individuals introduced from the wild. The blood samples were stored at −20 °C or −80 °C until DNA extraction. The muscle samples were stored in ethanol until DNA extraction. DNA was extracted from the samples using the QIAGEN DNeasy Blood and Tissue Kit (Qiagen, Hilden, Germany). The DNA concentration was measured using a NanoDrop instrument (Thermo Fisher Scientific, Waltham, MA, USA).

### 2.2. Short- and Long-Read Sequencing

To develop a modified reference genome, we used the individual that was employed to generate the draft genome in a previous study [2]. The isolated genomic DNA was used to construct short-insert libraries with TruSeq DNA PCR Free (350) (Illumina, San Diego, CA, USA), according to the standard protocols provided by Illumina. This was followed by 150 bp paired-end sequencing using a whole-genome shotgun sequencing strategy on an Illumina Hiseq X Ten platform. Library construction and sequencing took place at Macrogen (Kyoto, Japan). For long-read sequencing, the sequence library was developed using the Ligation Sequencing Kit (SQK-LSK109; Nanopore, Oxford, UK) with 1000 ng of DNA. Sequencing was performed using an R9.4.1 flow cell on a GridION device (Nanopore, Oxford, UK). Long-read sequencing took place at Bioengineering Lab. Co., Ltd. (Kanagawa, Japan).

### 2.3. Development of the Draft Genome of the TLC

The previous draft genome [2] was created using only short-read sequencing, while the modified draft genome was generated using a combination of short- and long-read sequencing.

Development of the modified draft genome of the TLC was carried out using Genomic Workbench ver. 20.02 (Qiagen). “Trim reads” was performed on all read data. Quality trimming was set at *p* = 0.1 and minimum length = 15 bp, and the read-through adapters were removed using “Illumina universal.” Furthermore, the “De novo assemble tool” was applied to the trimmed read data in the “Create simple contig sequence” mode using the following parameters: word size = 50, and minimum length = 500 (the remaining parameters were the default ones). The contig used in this step was “temporary contig A.”

Raw reads from the Nanopore sequencer were base called using Guppy_basecaller ver. 1.8.5. Passed reads were trimmed for adapter sequences using Porechop ver. 0.2.3. Trimmed reads were imported to Genomic Workbench ver. 20.02 as single reads. Subsequently, reads greater than 100 kb were removed. The contigs from short-read sequencing and the imported reads from long-read sequencing were combined using the “*Join contig*” program on Genomic Workbench ver. 20.02 (“temporary contig B”).

Trimmed reads were mapped to the domestic cat genome (GCF_000181335.3_Felis_catus_9.0_Genomic.fna) using “Map to reference” in the global alignment mode. Default values were used as parameters for the similarity fraction (0.9) and length fraction (0.5). Next, the contigs with more than 20× coverage were extracted from the mapping data using “Extract consensus sequences”; these contigs were termed “temporary contig C”.

The modified draft genome was created by joining “temporary contig B” and “temporary contig C” with “Joint contig”.

We assessed the gene completeness of our assemblies using Benchmarking Universal Single-Copy Orthologs (BUSCO) ver. 5 [17] with the Core Vertebrate Genes DB 10 BUSCO gene set through the gVolante (ver. 2.0.0) web server [18].

### 2.4. GRAS-Di Analysis

GRAS-Di analysis was used for DNA sequencing. This technology was developed by Toyota [19]. Library preparation and sequencing were carried out according to a previous study [2]. Library preparation and sequencing were performed by Bioengineering Lab. Co., Ltd.

Low-quality reads were trimmed using Sickle ver. 1.33 [20]. Nextera adapter and other Illumina primer sequences were clipped using default settings. A sliding window trimming step removed sequences with an average quality <30. Trimmed reads shorter than 50 bp were removed. Moreover, sequences after 50 bases were deleted using fastx_trimmer in FASTX-toolkit [21].

### 2.5. Genotyping

The SNP analysis was performed using Stacks version ver. 2.5 [22]. In the subsequent analysis, we added data from a previous study [2], in which the samples (*n* = 48) were derived from wild animals. Therefore, the total cohort comprised of 33 captive and 50 wild TLCs. We used the “ref_map pl” program and the modified draft TLC genome (DDBJ Accession Number: BIMV02000001-BIMV02049228) as the reference genome. This combination (“ref_map pl” program and reference genome) yielded the best results in the previous study [2].

Trimmed reads were mapped onto the modified TLC genome using BWA-mem [23]. The obtained SAM files were converted to BAM files and sorted using SAMtools ver. 1.4 [24,25]. The region with a coverage <5 was deleted by VariantBam ver.1.4.4 [26]. SNP calling for each individual was performed using the “ref_map pl” program of Stacks ver. 2.5. The “populations” program of Stacks ver. 2.5 was set as the default.

Plink ver. 2.0 [27] was used to filter the dataset to obtain SNPs found in at least 80% of the individuals (—geno 0.2) and to exclude individuals with more than 20% missing genotypes (—mind 0.2). In the subsequent quality-control step, the following parameters were used to filter low-confidence SNPs using PLINK ver. 2.0: Hardy–Weinberg Equilibrium (*HWE*) *p* < 0.05 and linkage disequilibrium between all possible pairs of loci in 76 individuals (29 captive and 47 wild individuals).

### 2.6. Analysis of Genetic Diversity

The output files from PLINK were converted to the Genepop format using PGDSpider ver. 2.1.1.5 [28]. The allelic richness (*Ar*) per locus was calculated using HP-Rare 1.1 [29]. The expected heterozygosity (*He*) and observed heterozygosity (*Ho*), probability of identity (*PID*), *PID* among siblings (*PID-sib*), and probability of exclusion (*PE*) (when the other parent is known) were calculated using GenALEx ver. 6.5 [30].

The SNP data were also analyzed with the structure program [31] in Strauto [32] using the admixture model to estimate the genetic structure of the population. We conducted an analysis with 10 iterations for each number of clusters (*K*, 1–10) and with Markov chain Monte Carlo sampling running for 500,000 samples with an initial burn-in of 300,000 samples. The *K* values described by Evanno et al. (2005) [33] and Pritchard et al. (2000) [31] were then calculated to identify the most reasonable *K* using the structure Harvester program [34]. Moreover, the most reasonable *K* values were identified using the parsimony index described by Wang (2019) [35]. Runs were visualized using Pophelper ver. 2.3.1. Population structure was also examined by performing a principal component analysis (PCA) using the R [36] package “adegenet” [37] to visualize the genetic relationship among different individuals in two dimensions.

## 3. Results

### 3.1. Development of the Draft Genome of the TLC

In this study, we obtained 131 Gb and 4.3 Gb from the short-read sequencing and the long-read sequencing, respectively. Together with the 137 Gb of short-read sequences used in the development of the previous draft genome, the draft genome of the TLC was improved. The developed assembly had a genome size of 2.422 Gb covered in 49,228 scaffolds with an average size and scaffold N50 of 49,196 bp and 214,455 bp, respectively (DDBJ Accession Number: BIMV02000001-BIMV02049228). The completeness assessment performed using BUSCO [17] revealed that 213 (91.42%) genes were complete, and 231 (99.14%) genes were “complete + Partial.”

### 3.2. Genetic Diversity

In total, 18,407,884 raw reads with an average of 557,815 reads per sample were obtained for 33 individuals via the GRAS-Di analysis (DDBJ Accession Number: DRR346528-DRR346570). The data obtained in this study and those from a previous study [2] were used in the subsequent analysis. Gras-Di analysis identified 926 candidate SNP markers by Stacks with default settings. After the filtering steps, the number of remaining individuals and loci were 139 loci and 76 individuals (29 captive and 47 in the wild).

The genetic diversity indices of the TLC in captivity and in the wild are summarized in Table 1. The *Ar* in the captive and wild populations was 1.67 and 1.82, respectively. The average *Ho* and *He* in the captive population for all loci were 0.065 (0.000–0.269) and 0.069 (0.000–0.233), respectively. The average (minimum value–maximum value) *Ho* and *He* in the wild population for all loci were 0.076 (0.000–0.209) and 0.074 (0.000–0.187), respectively. The differences between populations and between *He* and *Ho* were not significant. The cumulative *PID* values for all loci in the captive and wild populations were 9.8 × 10^−9^ and 7.2 × 10^−10^, respectively. The cumulative *PID-sib* values for all loci in the captive and wild populations were 1.1 × 10^−4^ and 3.0 × 10^−5^, respectively. The cumulative *PE* (when the other parent is known) for all loci in the captive and wild population was 0.9870 and 0.9934, respectively.

### 3.3. Genetic Structure within the TLC Populations

**Figure 2:**
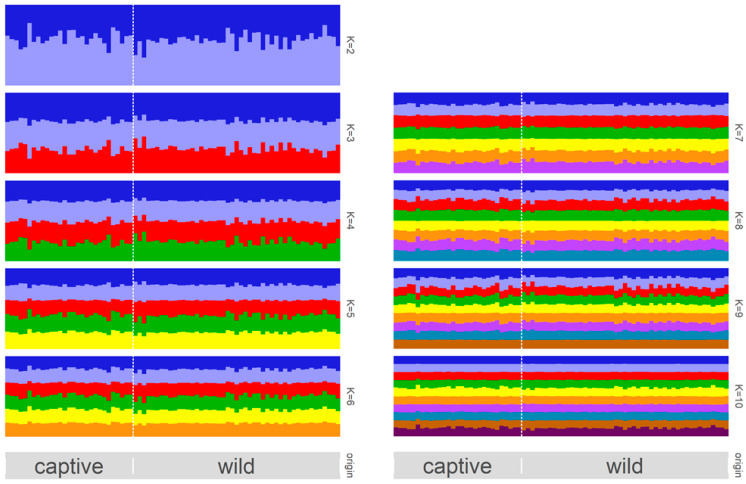
Bayesian analysis of the genetic structure of the populations, showing the differentiation of the captive and wild populations of the Tsushima leopard cat based on 139 SNPs. This figure was obtained using Pophelper to align the 10 replicates for optimal *K* = 21–10 (All runs were performed using the Markov chain Monte Carlo method running for 500,000 samples and using an initial burn-in of 300,000 samples).

**Figure 3:**
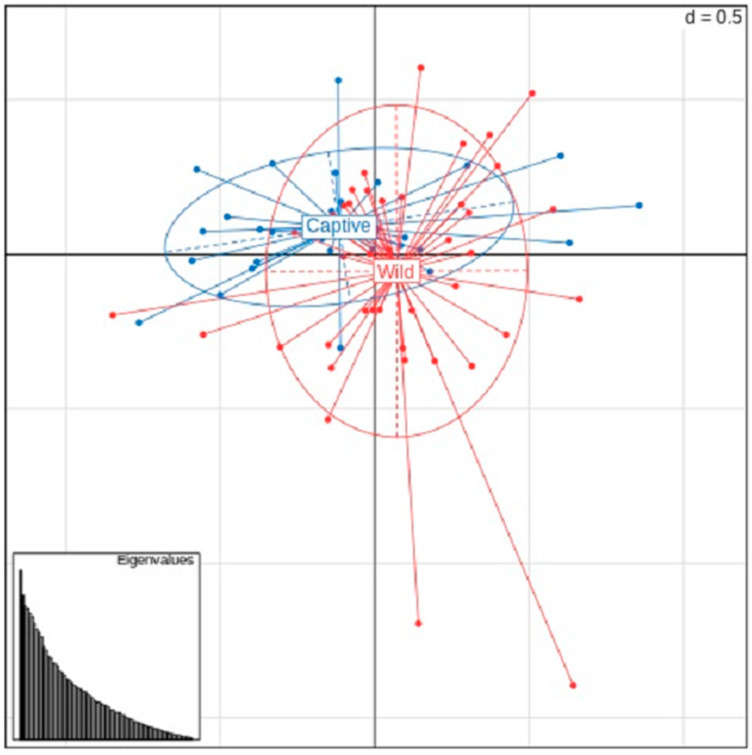
First and second components of the principal component analysis of 139 SNPs in the captive and wild populations of the Tsushima leopard cat. The percentages of variation explained by the first two axes were 4.97% and 4.24%, respectively. Each ellipse included 67% individuals from each population.

The most reasonable *K* values obtained based on deltaK, Ln Pr (X|K), and the parsimony index were 6, 4, and 1, respectively. The results of the structure analysis and PCA are presented in Figure 2 and Figure 3, respectively. The bar plots of each individual for *K* = 2–10 in the structure analysis are shown in Figure 2. In the PCA, most of the individuals were plotted in proximity. The percentages of variation explained by the first two axes were 4.97% and 4.24%, respectively (Figure 3).

## 4. Discussion

The modified draft genome generated in this study was more accurate than the previous draft genome generated using only short-read sequencing, with a 40% extension in scaffold N50 (152,598 to 214,455), a 32% extension in the average contig size (37,262 to 49,196), and a 25% reduction (65,356 to 49,228) in the number of contigs. These results pertaining to the length of scaffold N50 and the completeness assessment using BUSCO [17] were intermediate among the de novo assemblies of other carnivores [38]. However, in recent years, draft genomes have been generated mainly via long-read sequencing, which has greatly improved the scaffold N50 and the contig numbers. For a more detailed analysis, it is necessary to increase the number of analyses of long reads.

The number of SNPs obtained in the GRAS-Di analysis was 139, which was a decrease from the number analyzed in the wild population alone (*n* = 158). Compared to the previous study, it was expected that the number of SNPs would also increase due to the increase in the number of analyzed individuals and to the improvement in the accuracy of the draft genome, but the results were the opposite. This may be due to the low coverage of the samples analyzed in this study and an increase in the number of variants that did not meet the criteria (missing rate and/or HWE).

Although all indices of genetic diversity were slightly higher in the wild population than they were in the captive population, no difference between the two populations was considered. Although the captive populations are maintained in small groups, no differences in the genetic diversity indices were found between the wild and captive populations. The genetic diversity of the captive population was considered to be well-maintained, probably because the breeding population had a short history and some founder individuals had been introduced after the start of the breeding program. The captive breeding of the TLC began in 1996, with the reproductive effort (reproductive pairs were developed) beginning in 1999 and the first successful breeding occurring in 2000. The 31 samples analyzed in this study included five individuals from the wild and seven individuals from the first generation of captive breeding. Moreover, compared with the previous study [2], in which only wild populations were analyzed, all indicators of genetic diversity were lower. This may be attributed to the increase in the number of individuals to be analyzed and the decrease in the number of SNPs after filtering because of the decrease in average coverage. Because many SNP candidates are removed in the filtering step, more SNP markers can be expected to be developed due to the increased coverage. Filtering by deviations from the *HWE* may blur the significant deviations, such as those derived from evolutionary selection and deviations caused by genotyping errors. In this study, the coverage threshold was set to 5×, although it is desirable to set a higher coverage threshold for more accurate genotyping. Although the *PE* and *PID* were lower (worse) than those reported in the previous study, they may be useful for individual identification and parentage determination. Therefore, the SNP marker sets developed in this study can be applied to the elucidation of the ecology of the TLC, including the identification of individuals and the estimation of kinship, which is useful for the conservation of this species. However, because an increase in kinship caused by inbreeding is inevitable in small populations, it would be desirable to develop additional SNP markers to obtain accurate genetic information.

The best *K* values obtained in the structure analysis varied according to the three *K* calculation methods (*K* = 4 in Δ*K*, 6 in Ln Pr (X|K), and 1 in parsimony index). In the analysis of population structure, Δ*K*, Ln Pr (X|K), or both are often used to calculate the best *K* values. Because *ΔK* and Ln Pr (X|K) may overestimate or underestimate the value of *K* due to unbalanced sampling, low population differentiation, low marker information, hierarchical structure, and inbreeding [35,39], it is encouraged to present the plots of both Ln Pr (X|K) and Δ*K* or to include the structural bar plots of multiple *K* values [39]. The results of the bar plots generated here showed an absence of a clear population structure, not only at *K* = 4 and *K* = 6, which were considered the best values by Δ*K* and Ln Pr (X|K), but also at *K* = 2–10 (Figure 2). Because *K* = 1, which was shown to be the most reasonable *K* value in parsimony index, was accepted as being correct, no clear subpopulation was considered in the TLC population. Reportedly, the parsimony index is more suitable to calculate *K* than *ΔK* and Ln Pr (X|K) are under various conditions (Wang 2019). As it can be calculated from the output file of the structure software (Pritchard et al. 2000), as can Δ*K* and Ln Pr (X|K), we encourage the investigation of the parsimony index to clarify the best *K* values, together with Δ*K* and Ln Pr (X|K).

To clarify the genetic structure of a population, it is also recommended to compare the results of multiple methods (e.g., structure analysis with BAPS or PCA) [39]. In the PCA analysis, the ellipses containing 67% of the individuals in each of the wild and captive populations largely overlapped, and there were negligible differences between the two populations (Figure 2). Several individuals were plotted slightly apart from the ellipse. These outliers may be an effect of the low number of markers. Moreover, although the genetic differences were small and the explained variances (PC1, 4.97%; and PC2, 4.24%) were low, these individuals may possess a slightly different genetic composition. Therefore, the TLC is likely to be a high-priority individual for captive breeding. In most of the conservation programs for endangered species, the genetic relationships among the founders of captive populations are unknown; thus, the breeding plans are designed on the assumption that there is no kinship among the founders. For this reason, it has been pointed out that there is a difference between the genetic diversity obtained based on pedigree and genetic analyses [2]. Because of the small size of the population of wild TLCs, the genetic diversity observed may be lower than that detected based on pedigrees, and more careful breeding plans may be necessary. Moreover, the genetic diversity of captive populations may be lower, as individuals who have passed the reproductive age without descendants are included in the population.

## 5. Conclusions

This study revealed the genetic diversity of captive and wild TLCs. The results showed that the current captive population has the same level of genetic diversity as the wild population and is in appropriate condition. In addition, the present study identified the genetic relationships among captive individuals; therefore, these data will be useful when considering breeding plans for captive conservation.

## Figures and Tables

**Table 1:** Genetic diversity indices of two populations of Tsushima leopard cat.

	*N*	*Na*	*Ho*	*He*	*Ar*	*PE*	*PID*	*PID-Sib*
Captive	26.360	1.705	0.069	0.065	1.67	0.987	9.8 × 10^−9^	1.1 × 10^−4^
Wild	44.424	1.971	0.076	0.074	1.82	0.993	7.2 × 10^−10^	3.0 × 10^−5^

*N*: mean number of individuals genotyped, *Na*: the number of effective alleles, *Ho*: observed heterozygosity, *He*: expected heterozygosity, *Ar*: allelic richness, *PE*: Cumulative probability of exclusion, *PID*: Cumulative probability of identity, *PID-sib*: Cumulative *PID* among siblings.

## Data Availability

Data sharing is not applicable to this article.
